# Inhibition of HIF-1α Reduced Blood Brain Barrier Damage by Regulating MMP-2 and VEGF During Acute Cerebral Ischemia

**DOI:** 10.3389/fncel.2018.00288

**Published:** 2018-09-04

**Authors:** Yufei Shen, Jingxia Gu, Ziyun Liu, Congying Xu, Shuxia Qian, Xiaoling Zhang, Beiqun Zhou, Qiaobing Guan, Yanyun Sun, Yanping Wang, Xinchun Jin

**Affiliations:** ^1^Department of Neurology, The Second Affiliated Hospital of Jiaxing University, Jiaxing, China; ^2^Department of Neurology, The Second Hospital of Jiaxing City, Bengbu Medical College, Bengbu, China; ^3^Jiangsu Key Laboratory of Neuropsychiatric Diseases Research and Institute of Neuroscience, The Second Affiliated Hospital of Soochow University, Suzhou, China; ^4^School of Pharmacy, Key Laboratory of Molecular Pharmacology and Drug Evaluation, Ministry of Education, Yantai University, Yantai, China

**Keywords:** blood brain barrier, HIF-1α, ischemia stroke, matrix metalloproteinase, vascular endothelial growth factor

## Abstract

Increase of blood brain barrier (BBB) permeability after acute ischemia stroke is a predictor to intracerebral hemorrhage transformation (HT) for tissue plasminogen activator (tPA) thrombolysis and post-endovascular treatment. Previous studies showed that 2-h ischemia induced damage of BBB integrity and matrix metalloproteinase-2 (MMP-2) made major contribution to this disruption. A recent study showed that blocking β2-adrenergic receptor (β2-AR) alleviated ischemia-induced BBB injury by reducing hypoxia-inducible factor-1 alpha (HIF-1α) level. In this study, we sought to investigate the interaction of HIF-1α with MMP-2 and vascular endothelial growth factor (VEGF) in BBB injury after acute ischemia stroke. Rat suture middle cerebral artery occlusion (MCAO) model was used to mimic ischemia condition. Our results showed that ischemia produced BBB damage and MMP-2/9 upregulation was colocalized with Rhodamine-dextran leakage. Pretreatment with YC-1, a HIF-1α inhibitor, alleviated 2-h ischemia-induced BBB injury significantly accompanied by decrease of MMP-2 upregulation. In addition, YC-1 also prevented VEGF-induced BBB damage. Of note, VEGF was shown to be colocalized with neurons but not astrocytes. Taken together, BBB damage was reduced by inhibition of interaction of HIF-1α with MMP-2 and VEGF during acute cerebral ischemia. These findings provide mechanisms underlying BBB damage after acute ischemia stroke and may help reduce thrombolysis- and post-endovascular treatment-related cerebral hemorrhage.

## Introduction

Damage of blood brain barrier (BBB) integrity after acute ischemia stroke is a promising target (Jin et al., 2014; Liu et al., 2016) for clinical intervention to reduce hemorrhage transformation (HT) in patients with intravenous tissue plasminogen activator (tPA; Leigh et al., 2014) or post-endovascular treatment (Leigh et al., 2016). Most of studies focus on reperfusion-induced BBB damage because most of the damaging consequences of BBB injury (hemorrhage and edema) won’t be presented until the blood flow to the ischemic brain is restored (Simard et al., 2007; Hafez et al., 2014; Shi et al., 2016). However, BBB damage at the ischemia stage, particularly at the early stage of the 4.5 h thrombolytic time window (Hacke et al., 2008), remains a much less-well studied topic.

Recent studies showed that 2-h ischemia caused damage of BBB integrity in non-infarcted ventromedial striatum (Jin et al., 2012; Wang et al., 2016; Sun et al., 2017) and 2-h ischemia-induced matrix metalloproteinase-2 (MMP-2) induction lead to the disruption of the BBB integrity (Jin et al., 2012; Liu et al., 2012; Wang et al., 2016). In addition, blocking β2-adrenergic receptor (β2-AR) alleviated ischemia-induced BBB damage by reducing hypoxia-inducible factor-1 alpha (HIF-1α) level (Sun et al., 2017). However, it is not clear about the interaction of HIF-1α with MMP-2 in BBB damage during acute ischemia.

HIF-1α and its downstream vascular endothelial growth factor (VEGF) have been shown to play important role in the damage of BBB integrity after ischemia and reperfusion (Chen et al., 2009, 2010). VEGF has been shown to disrupt integrity of BBB by altering tight junction proteins under ischemic and inflammatory conditions (Yeh et al., 2007; Argaw et al., 2009; Engelhardt et al., 2014). However, the role of VEGF in BBB damage after acute ischemia as well as its cellular distribution is not known.

Therefore, in this study, using suture middle cerebral artery occlusion (MCAO) model to mimic ischemia stroke, we aimed to investigate the relationship of HIF-1α with MMP-2 and VEGF in BBB damage during acute cerebral ischemia. Our hypothesis is that acute ischemia stroke induced upregulation of HIF-1α and HIF-1α plays a critical role in BBB damage through interacting with its downstream MMP-2 and VEGF.

## Materials and Methods

### Animal Model of Focal Cerebral Ischemia

The Sprague-Dawley rats, which were ordered from SLAC Company (Shanghai, China), were housed 2–3 per cage under constant temperature (23 ± 1°C) and light-controlled vivarium (12-h light/12-h dark cycle). Food and water were available *ad libitum*. The animal procedures were approved by the University Committee on Animal Care of Soochow University and performed according to the NIH Guide for the Care and Use of Laboratory Animals. All efforts were made to reduce the number of animals and to minimize animal suffering. Rats with body weight from 270 g to 290 g were subjected to MCAO for 2 h using the suture model, as has been described previously (Liu et al., 2017). Briefly, rats were anesthetized with isoflurane (4% for induction, 1.75% for maintenance) during surgical procedures. Body temperature was maintained at 37.5 ± 0.5°C using a heating pad. The external carotid artery (ECA) and internal carotid artery (ICA) were exposed. A 4-0 silicone-coated monofilament nylon suture was inserted into the ICA via a cut on the ECA. Reperfusion was produced by gently withdrawing the suture out of the ECA. Successful surgery was further confirmed by tissue staining with 2,3,5-triphenyltetrazolium chloride (TTC).

### Rhodamine-Conjugated Dextran and Evan’s Blue Leakage Detection

Immediately after 2-h ischemia, Rhodamine-conjugated dextran (2,000 kDa, Invitrogen, Carlsbad, CA, USA) or Evan’s blue dye (EB; Sigma, St. Louis, MO, USA, 2% wt/vol in PBS, 3 mL/kg) was administered via the tail vein. All rats were reperfused for 10 min to allow sufficient circulation of Rhodamine-conjugated dextran or EB to the ischemic hemisphere (I), but minimize the effect of reperfusion on integrity of BBB. After reperfusion, the rat was transcardially perfused with ice-cold PBS followed by quickly taking the brain out (Liu et al., 2017). The EB maximally absorbs light of 620 nm and emits red fluorescence of 680 nm (Saria and Lundberg, 1983).

Rhodamine-conjugated dextran leakage was used to observe the co-localization of BBB injury. EB leakage was applied to check the co-localization of BBB injury and MMP activity or VEGF expression. Twenty-micrometer-thick cryosection was cut from the 8-mm-thick brain region as described (Jin et al., 2012) and mounted for fluorescence microscopy observation. BBB injury was visualized as leakage of Rhodamine-conjugated dextran or EB, which appeared as red fluorescence on brain sections. Brain sections were subjected to immunostain analysis for VEGF expression.

### YC-1 Administration

Rat was treated with 3-(5’-Hydroxymethyl-2’-furyl)-1-benzyl indazole (YC-1), the HIF-1α inhibitor (Cayman Chemical Company, Ann Arbor, MI, USA dissolved in a solution of 1% dimethyl sulfoxide, DMSO) which is known to downregulate HIF-1α at the post-translational level. In addition, YC-1 inactivates the COOH-terminal transactivation domain (CAD) of HIF-1α, and stimulates factor inhibiting HIF (FIH) binding, further suppressing HIF-1α (Dewitz et al., 2017). YC-1 was administered at 2 mg/kg body weight through femoral vein at 24 h and 30 min prior to the onset of ischemia. We chose the dose based on previous publication (Sun et al., 2017).

### Immunostaining

Immediately after 2-h ischemia, rat was perfused with ice-cold PBS followed by 4% PFA. Analysis of VEGF was carried out by immunostaining using the 20-μm-thick cryosection as described (Wang et al., 2017). In brief, tissue was pre-incubated for 1 h at room temperature in PBS containing 0.1% Triton X-100, 1% BSA, and 5% goat serum to block non-specific binding sites. Then anti-VEGF antibody (1:200, Abcam), anti-NeuN (1:200, Millipore), anti-GFAP (1:2,000, Millipore) primary antibody were applied to the sections and incubated at 4°C overnight. Cy3 conjugated secondary antibody (anti-mouse, 1:800) or 488-conjugated secondary antibody (anti-rabbit, 1:800) was incubated with the brain cryosection for 2 h at room temperature. The staining was visualized under LSM 700 confocal laser-scanning microscope (Zeiss), and images were taken from the ischemic and the mirrored non-ischemic region.

### Gel and *in situ* Gelatin Zymography

#### *In situ* Zymography

*In situ* zymography was used to check the gelatinolytic activity of MMP-2/9 in brain tissue by using Kit (EnzCheck Collagenase Kit, Invitrogen) following the manufacturer’s instructions as described (Shu et al., 2015). Rat brain was sliced into 20-μm-thick cryosections and cryosections were incubated in a reaction buffer which contained 30 μg/ml of FITC-labeled DQ-gelatin in a humidity chamber for 1 h at 37°C. The sections were rinsed and mounted for fluorescent microscopic observation. The disruption of BBB integrity was reflected by Rhodamine dextran or EB extravasation (red), and the gelatin-FITC is cleaved by gelatinases, yielding peptides whose fluorescence is representative of net proteolytic activity (green).

### Gel Gelatin Zymography

Tissues from ischemic (I) and non-ischemic (NI) hemisphere were homogenized in matrix metalloproteinase lysis buffer, and levels of MMP-2/9 in the homogenates were detected by gel gelatin zymography as described previously (Sun et al., 2017). A mixture of human MMP-2/9 (Invitrogen) was used as a positive control (gelatinase standards).

### Statistical Analysis

The data are showed as mean ± SEM. Statistical analysis was done using one way or two-way ANOVA (SPSS software, version 17.0). A value of *P* < 0.05 was considered statistically significant.

## Results

### Effect of 2-h Ischemia on BBB Damage and MMP-2/9 Activity

**Figure 1:**
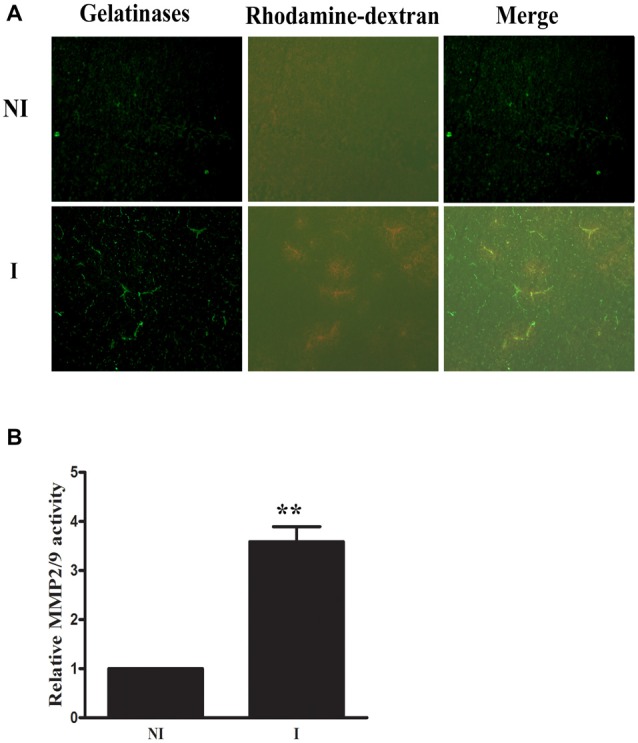
Effect of 2-h ischemia on blood brain barrier (BBB) damage and matrix metalloproteinase-2 (MMP-2)/9 activity. **(A)** After *in situ* zymography procedure, ischemic region (I) with dextran leakage (bottom left panel) and its corresponding tissue (upper left panel) in the non-ischemic hemisphere (NI) were chosen for microscopic observation. Fluorescent micrographs showed increased gelatinolytic activity of MMP-2/9 along ischemic microvessels (bright green fluorescence), where dextran leakage concurrently occurred. Spotted distribution of increased gelatinolytic activity was also seen in the leaky area. No dextran leakage and weak gelatinolytic activity were seen in the corresponding region of the NI hemisphere. **(B)** Quantitative data of MMP-2/9 activity (*n* = 3). **P < 0.01 compared to MMP2/9 activity in NI hemisphere.

Rhodamine-dextran leakage which showed BBB damage was clearly seen in the ischemic area (Figure 1). *In situ* zymography was done to check the spatial distribution of gelatinase activation and its colocalization with 2-h ischemia-induced BBB injury. After incubating the brain slice with FITC-labeled DQ gelatin, compared to the non-ischemic (NI) hemisphere, there was a significant increase of gelatinolytic activity (green fluorescence) in the ischemic (I) hemisphere where dextran leakage was detected (Figure 1A) and quantitative data confirmed the ischemia-induced MMP-2/9 activity upregulation (Figure 1B). Remarkably, more gelatinolytic activities were found with the leaked ischemic microvessels (Figure 1A).

### MMP-2 Induction After 2-h Ischemia

**Figure 2:**
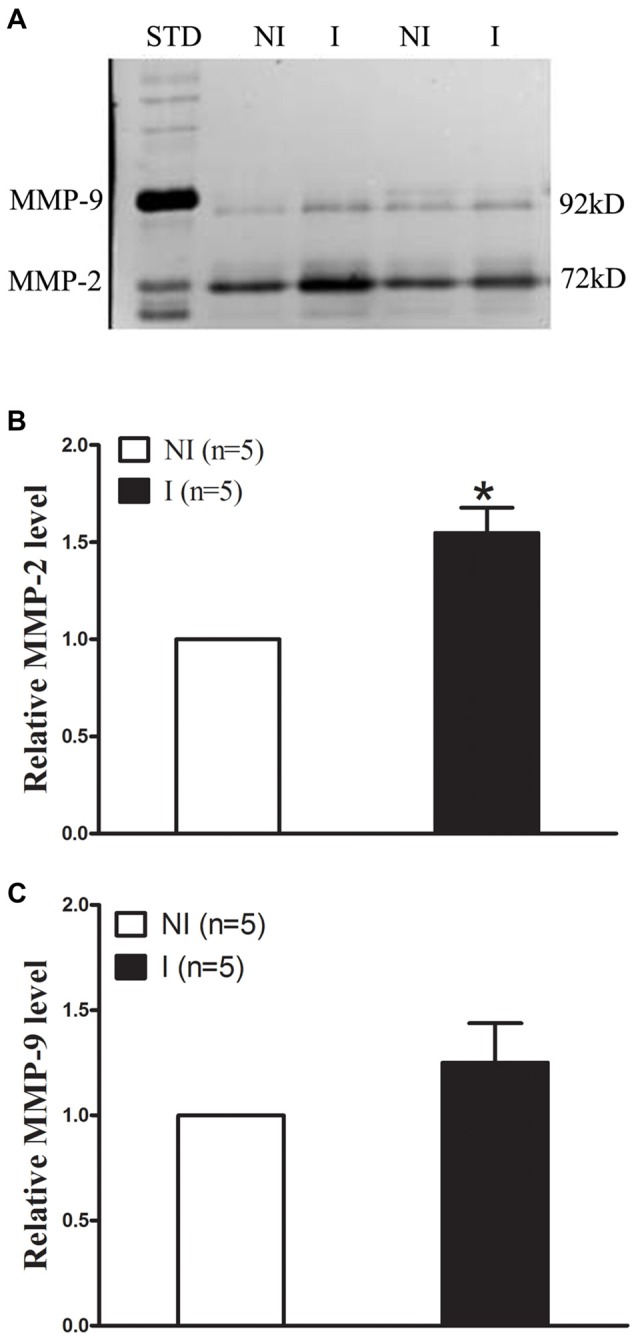
Effect of 2-h ischemia on MMP-2/9 induction. MMP-2/9 was induced in ischemic brain tissue after 2-h ischemia. **(A)** Representative gelatin zymogram showed MMP-2/9 levels in the non-ischemic (NI) and ischemic (I) hemispheric tissue. MMP-2 bands were much stronger than MMP-9 bands on zymogram gels. STD is a mixture of standard MMP-2/9. The relative band intensity of MMP-2 **(B)** and MMP-9 **(C)** was quantified. A significant increase was observed for MMP-2 in the ischemic tissue after 2-h middle cerebral artery occlusion (MCAO; *P < 0.05 vs. NI, *n* = 5), while there was no significant increase for MMP-9 (*P* > 0.05 vs. NI, *n* = 5). Data were expressed as mean ± SEM.

The levels of MMP-2/9 were analyzed with gel zymography after 2-h of ischemia (Figure 2). There is no significant difference between the levels of MMP-2 in the non-ischemic (NI) hemisphere and similar results were observed for MMP-9 (Figure 2A). The level of MMP-2 was significantly increased after 2-h ischemia (Figure 2B), while the level of MMP-9 was much lower and there is no significant increase (Figure 2C).

### HIF-1α Inhibitor YC-1 Alleviated 2-h Ischemia-Induced BBB Disruption as Well as MMP-2 Upregulation

**Figure 3:**
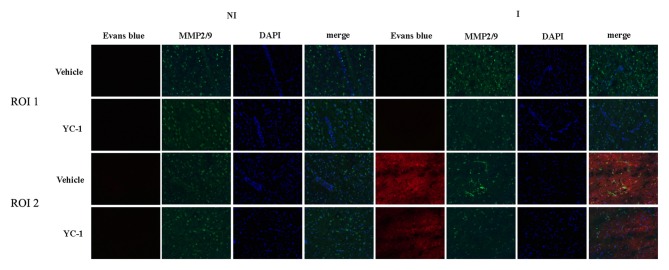
Effect of 3-(5’-Hydroxymethyl-2’-furyl)-1-benzyl indazole (YC-1) treatment on 2-h ischemia-induced BBB damage and MMP-2 induction. Rats received YC-1 at 24 h and 30 min before the onset of ischemia. Evan’s blue (EB) leakage (red) was seen in the ischemic region of interest 2 (ROI 2) after 2-h ischemia and *in situ* zymmography showed increased MMP-2 activity in the brain area where EB leakage occurred. Pretreatment with YC-1 significantly prevented MMP-2 induction as well as EB leakage. *n* = 3/group.

EB leakage was often used to evaluate the integrity of BBB (Wang et al., 2017). After 2-h ischemia, there was an obvious EB leakage in the ipsilateral hemisphere of rat brain (Figure 3). HIF-1α is a key mediator of the adaptive cellular response to hypoxia condition. To determine the interaction HIF-1α and MMP-2 in BBB damage after 2-h ischemia, treatment with HIF-1α inhibitor YC-1 dramatically reduced the EB leakage, indicating that HIF-1α inhibition with YC-1 protected BBB against 2-h ischemia-induced damage. Using *in situ* zymography, our results showed that MMP-2 was significantly increased in region of interest 2 (ROI 2) and a colocalization was observed for MMP-2 and EB leakage. YC-1 administration significantly decreased BBB damage as well as MMP-2 activity (Figure 3).

### Effect of HIF-1α Inhibition on Colocalization of VEGF With EB Leakage

**Figure 4:**
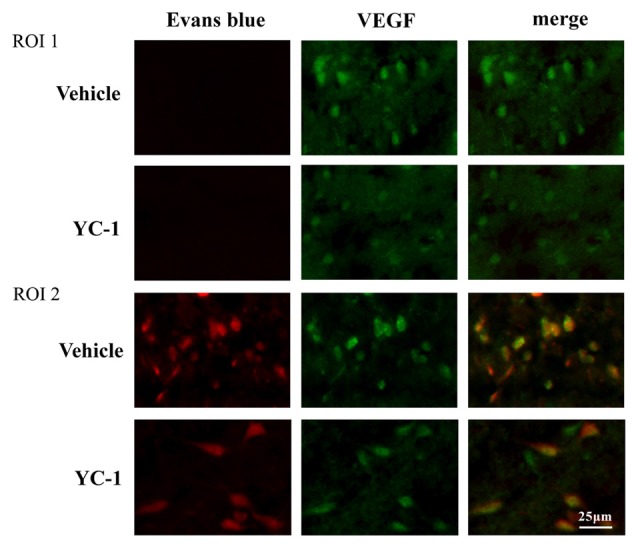
Effect of hypoxia-inducible factor-1 alpha (HIF-1α) inhibition on colocalizaion of vascular endothelial growth factor (VEGF) with EB leakage. Representative fluorescent micrographs of EB leakage (red) and VEGF expression in non-ischemic (NI) and ischemic site (I). Two-hour MCAO induced significant EB leakage (BBB damage) in ROI 2 of ischemic site and inhibition of HIF-1α with YC-1 significantly decreased EB leakage. *n* = 4.

VEGF has been implicated in BBB permeability increase (Schoch et al., 2002; Yan et al., 2011). To check the interaction of HIF-1α and VEGF in 2-h ischemia-induced BBB injury, immunofluoresence was performed to determine the spatial distribution of VEGF and its co-localization with BBB damage. After 2-h ischemia, VEGF (green fluorescence) was seen in the ischemic hemisphere (ROI 2) where EB leakage was observed (red), there is a co-localization of VEGF and EB leakage (Figure 4). YC-1 pretreatment significantly decreased the VEGF as well as decreased the BBB leakage (Figure 4).

### Colocalization of VEGF With Neurons and Astrocytes

**Figure 5:**
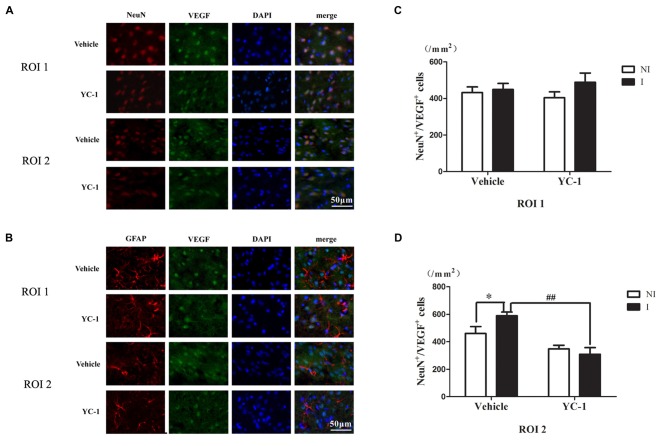
Effect of YC-1 on 2-h ischemia-induced VEGF expression in neurons and astrocytes. The cellular distribution of VEGF was analyzed by immunostaining with NeuN (marker of neurons) or GFAP (marker of astrocytes) after 2-h MCAO. Double immunostain of VEGF (green) and NeuN (red) showed a good colocalization of VEGF and neurons and YC-1 treatment significantly decreased the proportion of VEGF-positive neurons **(A)**. Double immunostain of VEGF (green) and GFAP (red) showed no co-localization of VEGF and astrocytes **(B)**. After quantification, two-way ANNOVA showed an increased proportion of VEGF positive neurons and YC-1 significantly decreased this upregulation **(C,D)**. *n* = 3/group. Scale bar = 50 μm. *n* = 3/group. *P < 0.05 compared to the NeuN+/VEGF+ cells in the NI hemisphere. ^##^*P* < 0.01 compared to NeuN+/VEGF+ cells in I hemisphere of Vehicle group.

A cell type-specific reaction for HIF-1α within endothelial cells, astrocytes and neurons after 2-h ischemia stroke has been defined (Sun et al., 2017). In addition, it has been reported that there were differential changes of glutathione levels in astrocytes and neurons in ischemic hemisphere (Bragin et al., 2010). However, the cellular distribution of VEGF after acute ischemia stroke was not known. Double-immunostaining was applied to check the cellular distribution of VEGF and the nuclear localization was confirmed by colocalization with DAPI staining. As shown in Figure 5, VEGF was mainly localized in neurons (Figure 5A), but not in astrocytes (Figure 5B) in the ROI 2 after 2-h ischemia. YC-1 significantly downregulated the proportion of VEGF-positive neurons (Figures 5C,D). Therefore, it was neuron that continuously produced VEGF after 2-h ischemia.

## Discussion

The integrity of BBB after acute ischemia stroke is important for determining the efficacy of thrombolysis with tPA (Leigh et al., 2014) or post-endovascular treatment (Leigh et al., 2016). This has motivated us to study early ischemic BBB injury, with an attempt to improve acute stroke management (the critical time window for rescuing ischemic neurons) in the long run and give some clue for future tPA thrombolysis. In our current study, we showed that: (1) ischemia damaged BBB integrity and MMP-2/9 upregulation was colocalized with Rhodamine-dextran leakage. (2) Pretreatment with YC-1, a HIF-1α inhibitor, significantly alleviated 2-h ischemia-induced BBB damage accompanied by inhibition of MMP-2 upregulation. In addition, inhibition of HIF-1α prevented VEGF-induced BBB damage. (3) VEGF was shown to be colocalized with neurons but not astrocytes. Taken together, BBB damage was reduced by inhibition of interaction of HIF-1α with MMP-2 and VEGF during acute cerebral ischemia.

Interaction of HIF-1α with MMP-2 and VEGF has been shown to play a critical role in barrier integrity. For example, pharmacological inhibition of HIF-1α by YC-1 markedly suppressed the expression of HIF-1α, VEGF and MMP-2, mitigated the severity of BBB disruption and attenuated isoflurane-induced cognitive deficits in the Morris water maze task in aged rat model of postoperative cognitive dysfunction (Cao et al., 2018).

MMP-2/9 has been shown to play important role in BBB damage at the stage of reperfusion (Liu and Rosenberg, 2005; Yang et al., 2007; Jin et al., 2013) and at acute ischemia stage (Jin et al., 2012; Wang et al., 2016). Previous study showed that 2-h MCAO induced BBB damage through upregulating HIF-1α which appeared to be mostly induced in the non-infarcted ventral striatum and preoptic area (Sun et al., 2017). Consistent with previous study showing that HIF-1α is implicated in the control of MMP (Jalal et al., 2015), our current study provide evidence that MMP-2 activity was significantly increased in ventral striatum and preoptic area, interaction of HIF-1α with MMP-2 play important role in BBB damage during acute ischemia and inhibition of HIF-1α could reduce BBB damage through regulating MMP-2 activity. Thus, HIF-1α may be a promising target for reducing BBB damage after acute ischemia stroke.

In our current study, we showed that VEGF played critical role in BBB damage during acute ischemia which was consist with previous studies showing that VEGF is involved in BBB permeability increase after ischemia stroke (Schoch et al., 2002; Argaw et al., 2009; Yan et al., 2011). In addition, HIF-1α-VEGF-mediated tight junction dysfunction has been shown in choriocarcinoma cells (Zhang et al., 2017) and HIF-1α-VEGF signaling has been shown to play an important role in BBB protection against ischemia-reperfusion-induced injury (Yeh et al., 2007; Yan et al., 2011). For example, inhibition of HIF-1α and VEGF reduced HT in the ischemic brain which was induced by acute hyperglycemia (Chen et al., 2010; Zhang et al., 2016) and inhibition of HIF-1α-VEGF signaling reduced BBB damage in rat neonatal stroke model (Mu et al., 2003).

Of note, hypoxic Müller cells-secreted VEGF increased MMP-2 activity in endothelial cells (Rodrigues et al., 2013). In addition, early VEGF inhibition attenuated BBB disruption in ischemic rat brains by regulating the expression of MMPs (Zhang et al., 2017) and VEGF-MMP pathway played important role in progesterone’s protective effect on delayed tPA treatment-induced hemorrhagic transformation (Won et al., 2014). Therefore, interaction of HIF-1α with MMP-2 and VEGF play an important role in BBB damage.

Our result that VEGF was colocalized with neurons but not astrocytes is consistent with previous study showing that HIF-1α was co-localized with neurons but not astrocytes or endothelial cells (Sun et al., 2017). Astrocytes have the ability to be resistant to hypoxic condition and severe oxygen deprivation was needed to activate HIF-1α signaling pathway and regulate survival and proliferation in astrocytes (Schmid-Brunclik et al., 2008). Two-h ischemia may not be serious enough for astrocytes to produce VEGF. However, with duration of ischemia was extended, astrocytes played more important role in BBB damage, for example, Li et al. (2014) showed that 3-h ischemia facilitated neurons to activate astrocytes to damage barrier of endothelial cells via increasing expression of VEGF.

Nitric oxide (NO)-mediated many conditions can lead to disruption of BBB integrity, eventually leading to vasogenic edema and secondary brain damage (Gu et al., 2012). One of the major actions of NO is activation of soluble guanylate cyclase (sGC) resulting in generation of cGMP (Lamothe et al., 2004). YC-1, a dual functioning chemical, functions as both an inhibitor of HIF-1α and a direct activator of sGC (Huh et al., 2011). For example, YC-1 has been shown to protect white matter axons from NO toxicity and metabolic stress (Garthwaite et al., 2002). The effect of YC-1 on ischemia-induced BBB damage may be through modulation of NO. However, it has been previously shown that 2-h ischemia induced occludin degradation, but not claudin-5 degradation, although 2-h OGD increased NO, only 4-h OGD induced NO-dependent claudin-5 degradation (Liu et al., 2016). In our current study, 2-h ischemia induced BBB damage and this disruption is independent of NO (Gu et al., 2012), excluding the possibility that YC-1 affect the BBB integrity through modulating NO.

In summary, the findings may give new insights to prevent the BBB from ischemic damage and to extend the time window of tPA treatment or endovascular treatment and alleviate HT.

## Author Contributions

The work was performed and accomplished by all authors. YS, JG, ZL, CX, SQ, XZ, BZ, YS and QG contributed to the execution of the entire project and the statistical analyses. YS, JG, XJ and YW wrote the manuscript. All authors have read and approved the final manuscript.

## Conflict of Interest Statement

The authors declare that the research was conducted in the absence of any commercial or financial relationships that could be construed as a potential conflict of interest.

## Abbreviations

BBB: blood brain barrier
β2-AR: β2-adrenergic receptor
EB: Evan’s blue dye
ECA: external carotid artery
GSH: glutathione
HIF-1α: hypoxia-inducible factor-1 alpha
HT: hemorrhagic transformation
I: ischemic hemisphere
ICA: internal carotid artery
MCAO: middle cerebral artery occlusion
MMP: matrix metalloproteinase
NI: non-ischemic
NO: nitric oxide
ROI: region of interest
sGC: soluble guanylate cyclase
tPA: tissue plasminogen activator
TTC: 2,3,5-triphenyltetrazolium chloride
VEGF: vascular endothelial growth factor.
